# Supplementary material for: Identification and Characterization of Three New Antimicrobial Peptides from the Marine Mollusk Nerita versicolor (Gmelin, 1791)
Source: Int J Mol Sci. 2023 Feb 14;24(4):3852. doi: 10.3390/ijms24043852 (PMC9968088; doi:10.3390/ijms24043852)
Supplement: Supplementary file 1 [file ijms-24-03852-s001.zip › Figure S1.pptx]

## Slide 1
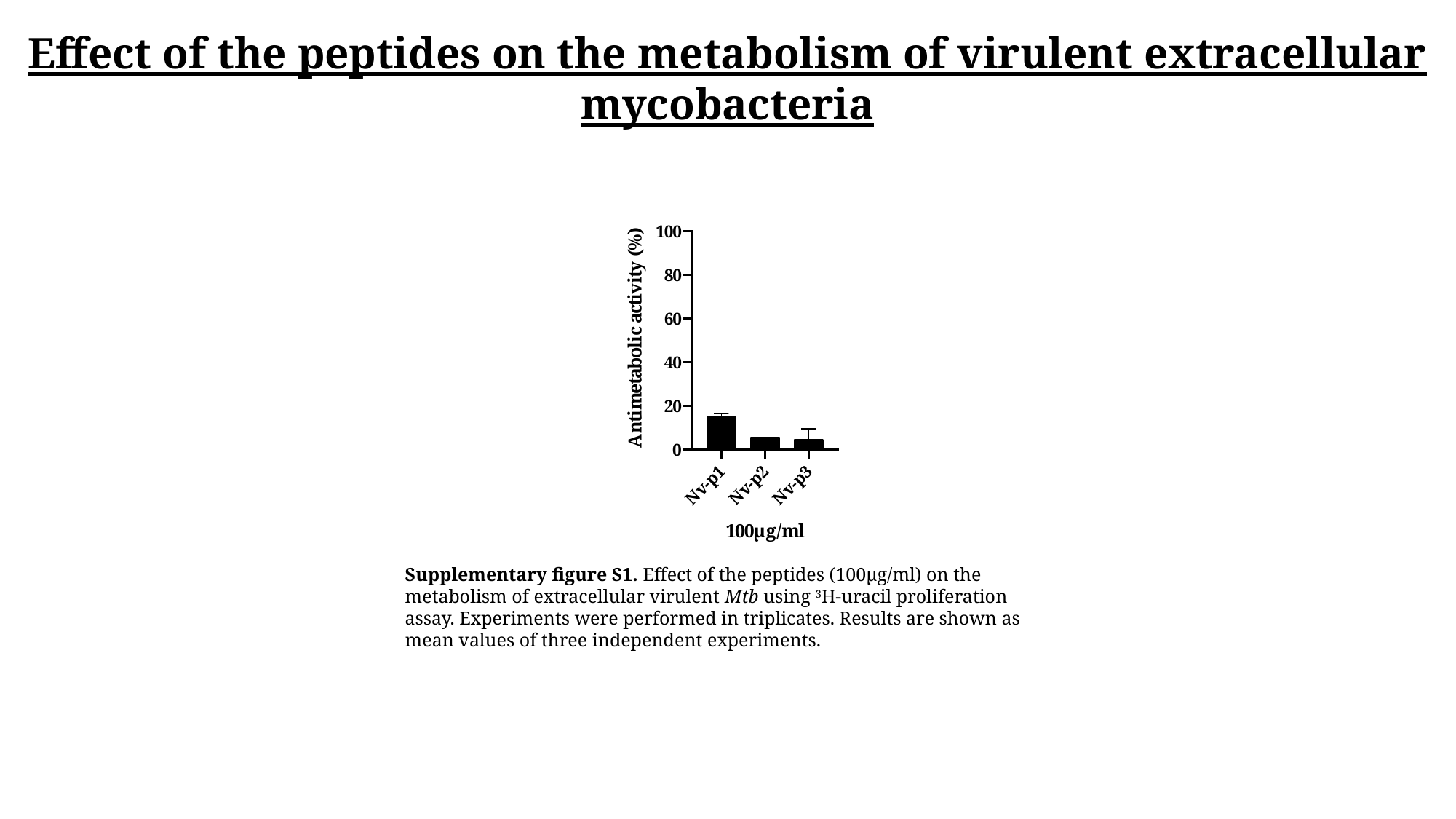

Effect of the peptides on the metabolism of virulent extracellular mycobacteria
Supplementary figure S1. Effect of the peptides (100µg/ml) on the metabolism of extracellular virulent Mtb using 3H-uracil proliferation assay. Experiments were performed in triplicates. Results are shown as mean values of three independent experiments.
