# Supplementary material for: Identification and Characterization of Three New Antimicrobial Peptides from the Marine Mollusk Nerita versicolor (Gmelin, 1791)
Source: Int J Mol Sci. 2023 Feb 14;24(4):3852. doi: 10.3390/ijms24043852 (PMC9968088; doi:10.3390/ijms24043852)
Supplement: Supplementary file 1 [file ijms-24-03852-s001.zip › Supl. Analytical data Nv-p1 to Nv-p3.pdf]

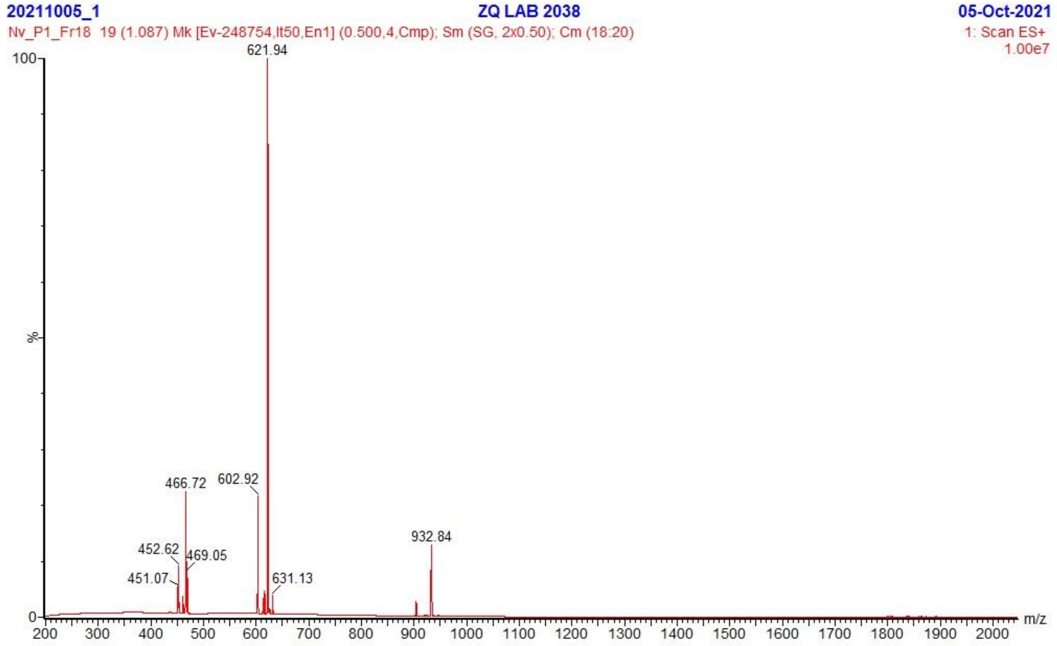


ESI-MS spectra of the synthetic Nv-p1, the 4+, 3+ and 2+ ion species have been detected.

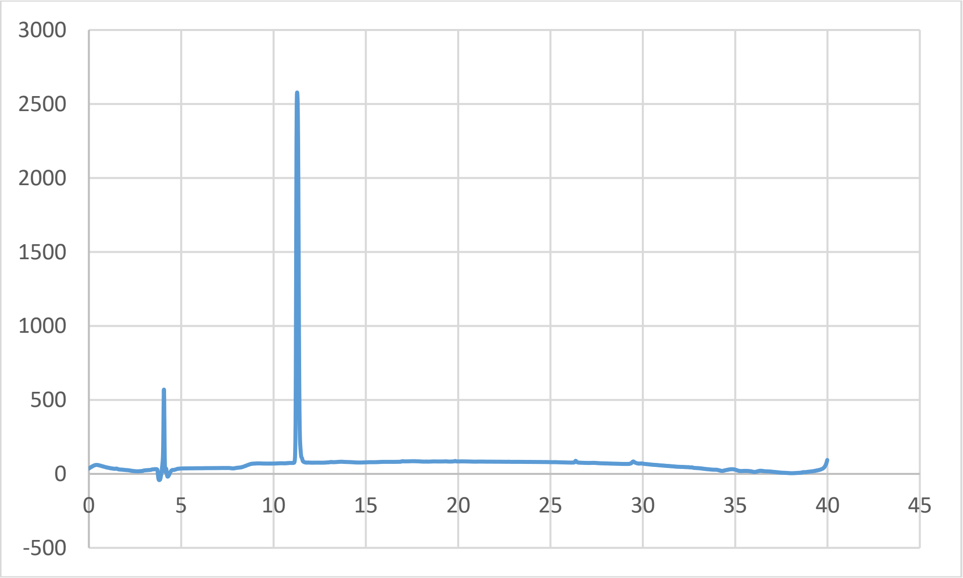

Analytical RP-HPLC of synthetic Nv-p1.

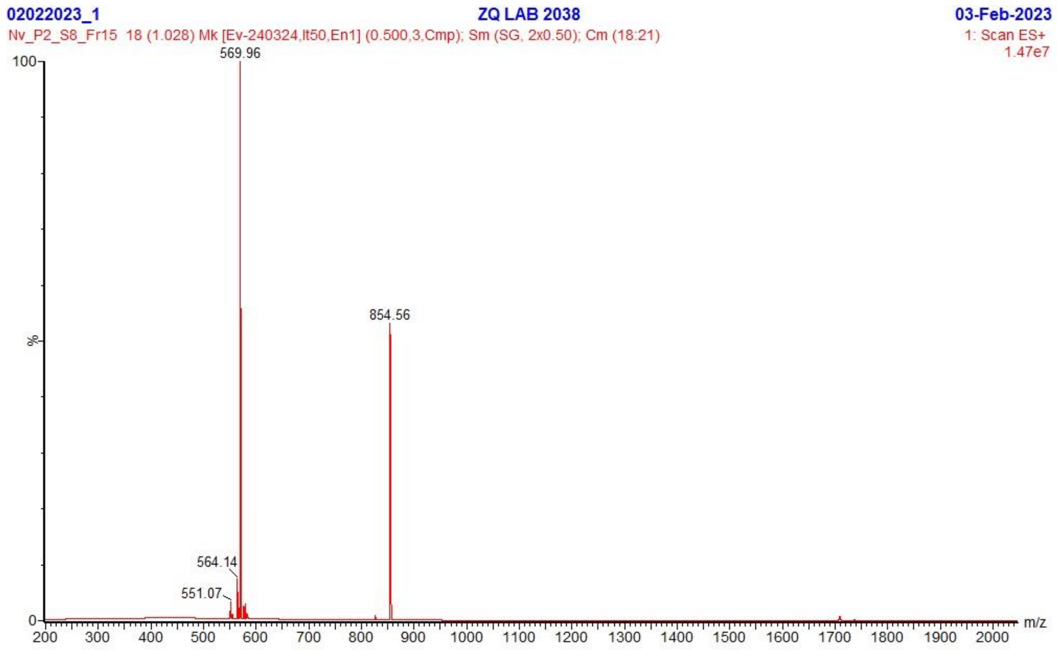


ESI-MS spectra of the synthetic Nv-p2, the 3+ and 2+ ion species have been detected.

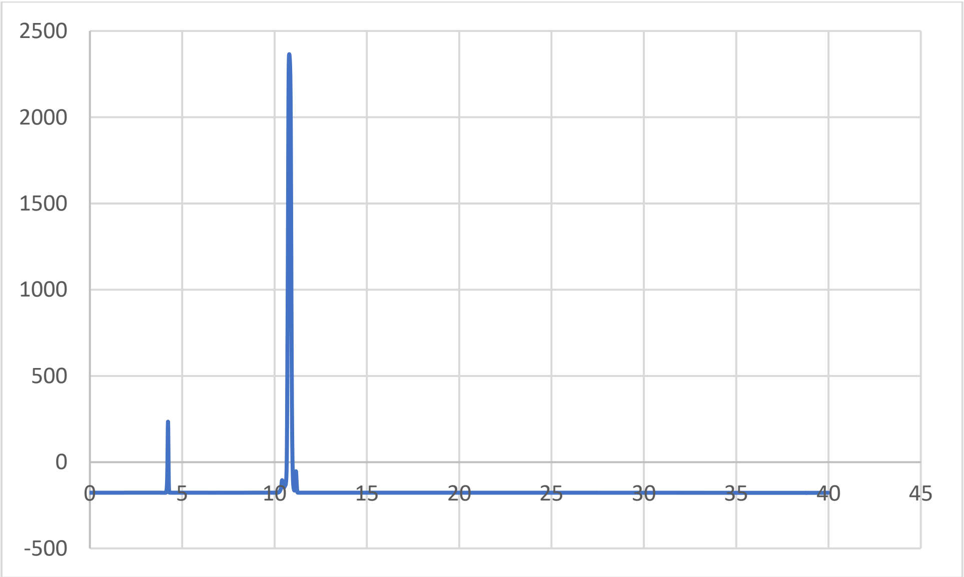


Analytical RP-HPLC of synthetic Nv-p2.

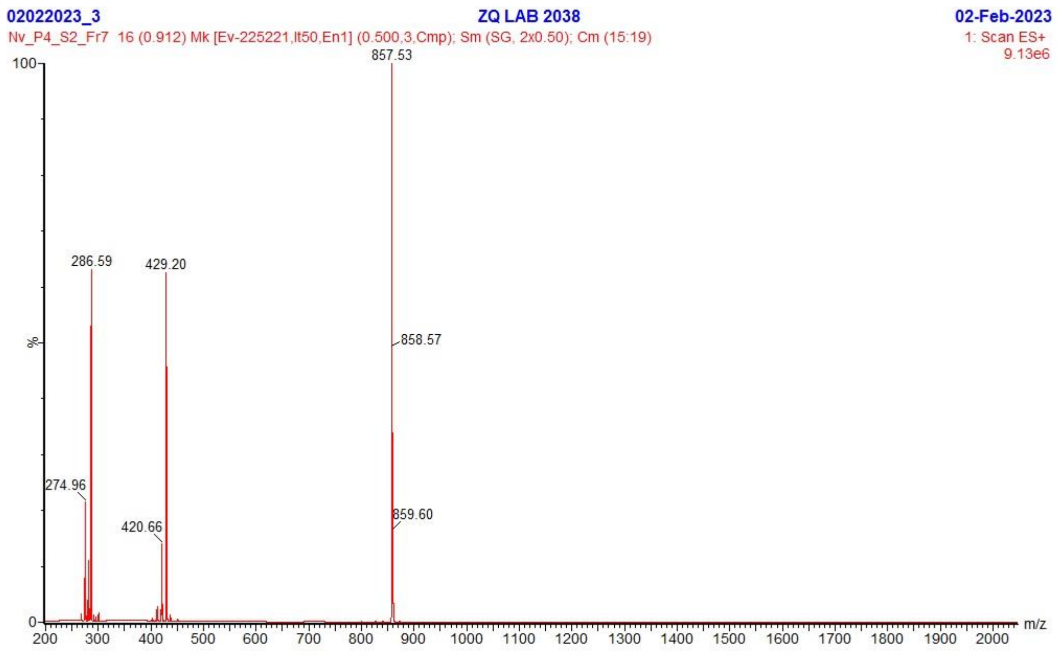


ESI-MS spectra of the synthetic Nv-p3, the 3+, 2+ and 1+ ion species have been detected.

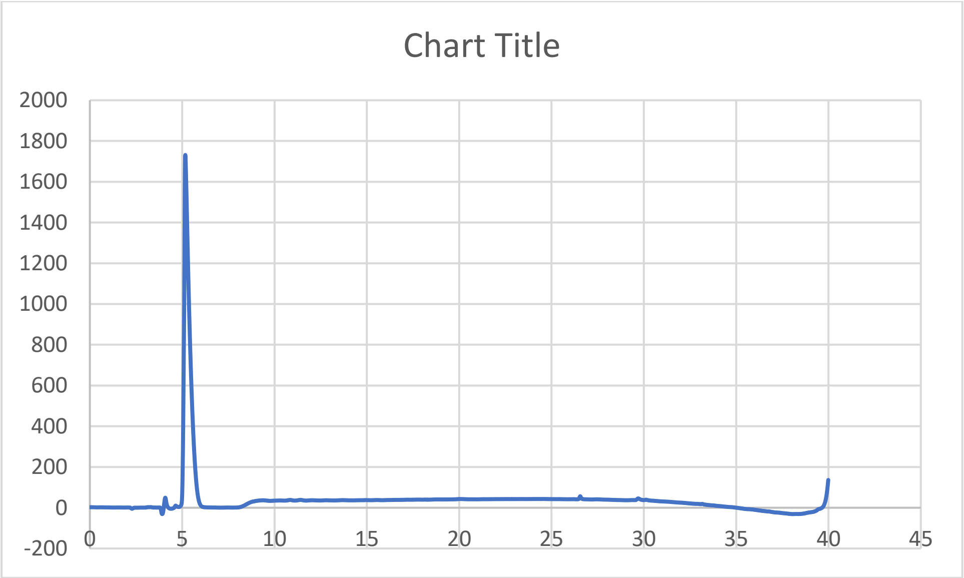


Analytical RP-HPLC of synthetic Nv-p3.
